# An Integrated Evanescent Field Sensor for the Simultaneous Measurement of Layer Refractive Index and Thickness

**DOI:** 10.3390/s21051628

**Published:** 2021-02-26

**Authors:** Matthias Jäger, Jürgen Bruns, Jessica Schneidewind, Cay Pinnow, Hassan Gargouri, Klaus Petermann

**Affiliations:** 1 Fachgebiet Hochfrequenztechnik/Photonik, HFT4, Technische Universität Berlin, 10587 Berlin, Germany; matthias.jaeger@tu-berlin.de (M.J.); petermann@tu-berlin.de (K.P.); 2 Plasma Process Technology Group, SENTECH Instruments GmbH, 12489 Berlin, Germany; jessica.schneidewind@sentech.de (J.S.); cay.pinnow@web.de (C.P.); hassan.gargouri@googlemail.com (H.G.)

**Keywords:** silicon photonics, silicon on insulator (SOI), ring resonator, refractive index sensing

## Abstract

A novel integrated sensor for the simultaneous measurement of layer refractive index and thickness based on evanescent fields is proposed. The theoretical limits for the accuracy of the sensor were examined for the example of a ${\mathrm{TiO}}_{2}$ layer. The influence of production tolerance on the accuracy was evaluated. In the experimental part of this work, a sensor chip containing nanowire and nanorib waveguides realized in silicon on insulator technology was used to demonstrate the detection of refractive index and thickness of a ${\mathrm{TiO}}_{2}$ atomic layer deposition (ALD) layer.

## 1. Introduction

Waveguide-based evanescent field sensing is a growing field of interest in integrated optics with applications in many areas, e.g., label-free biosensing and the detection of chemicals [1,2,3,4,5]. A waveguide small enough for its fundamental mode to have a significant evanescent field outside the waveguide core allow for light–matter interaction at the waveguide’s surface. Changes of the refractive index in the volume penetrated by the evanescent field lead to changes in the effective index $n_{eff}$ of the light traveling in the waveguide. Optical structures such as ring resonators [6] or Mach–Zehnder interferometers [7,8] can be used to translate such a change into a shift of a resonance wavelength. This shift can easily be measured by detecting the transmission spectrum of such a device.

Microring resonators based on silicon on insulator (SOI) sensing technology have gathered a lot of attention so far, mainly in the field of label-free optical biosensing [9,10]. The high-refractive index contrast between waveguide and cladding layers allows for very small structures to be processed on silicon microchips and thus enables large-scale production at low cost [11]. Moreover, CMOS-compatible processing makes them suitable for photonic-electronic co-integration [12].

One limitation of layer or liquid property measurements using these sensor systems is the inherent spatial integration during the measurement. The measurement is not only averaged along the length of the waveguide but forms a weighted average over the volume accessible to the evanescent field of the waveguide. This leads to an ambiguity where a layer of a given height and refractive index cannot be distinguished from a thinner layer with a larger refractive index.

In applications such as biosensing, this is not a limitation, because only a relative shift of a resonance wavelength is detected. For a more detailed investigation of solid layers or liquids, the simultaneous retrieval of layer thickness and refractive index is necessary. One approach to accomplish this is the use of two different states of polarization during the measurements. In [13,14], a successful demonstration of the monitoring of refractive index and layer thickness is given. Using this technique, the simultaneous measurement of the resonance wavelength shifts for transverse electric (TE) and transverse magnetic (TM) modes is necessary.

**Figure 1:**
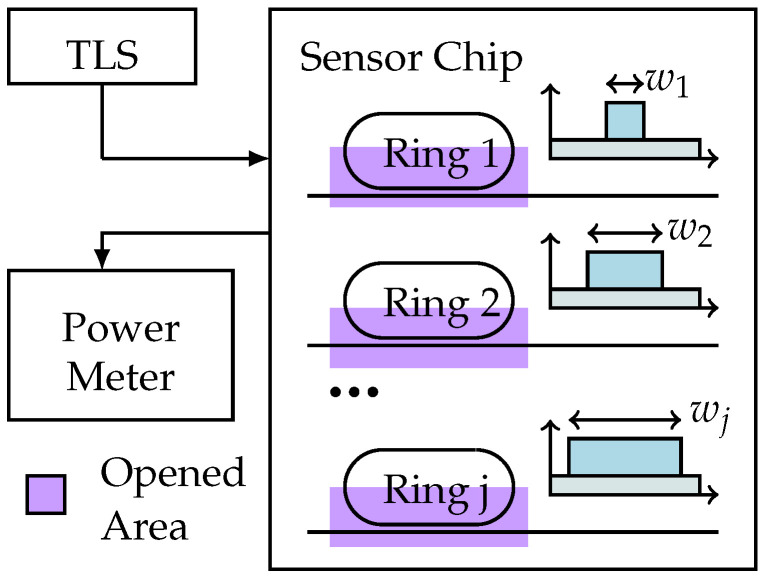
Sketch of the measurement system with the sensor chip. A tunable laser source (TLS) is used to sweep through the desired wavelength range; a power meter records the transmission spectrum. On the sensor chips, *j* individual ring resonators coupled to access waveguides are used for the measurements. In our realization, the sensor chip was covered by a protective ${\mathrm{SiO}}_{2}$ layer. This protective layer was etched away for half of the area of the ring resonator (marked in purple). Only this area was accessible to the sample layer.

In this paper a novel sensor system using a different approach is presented. It combines multiple ring resonators formed by waveguides of different dimensions (see Figure 1), using a single TE polarization. The penetration depths of the evanescent field depend on the waveguide geometry. By combining different geometries of a few waveguides, it is possible to spatially resolve the refractive index and thickness of a layer. Due to the difference in penetration depth, the effective index of each waveguide and thereby the resonance wavelength of each resonator react differently to changes in sample layer thickness and refractive index.

## 2. Materials and Methods

### 2.1. Waveguide Geometries

**Figure 2:**
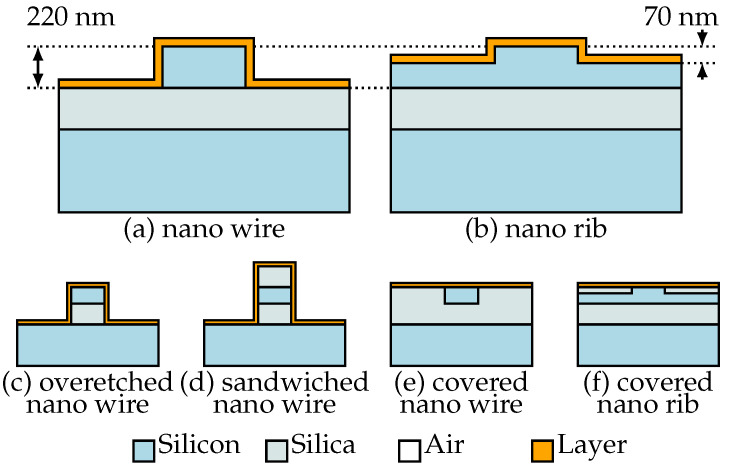
Sketches of the applied (**a**,**b**) and proposed (**c**–**f**) waveguide geometries.

Two different basic types of SOI-waveguides were considered for the ring resonator sensors. These included nanowire (Figure 2a) and nanorib waveguides (Figure 2b), both etched into the 220nm top silicon layer of an SOI wafer. The etch depth of the nanorib waveguides was 70nm.

For the theoretical analysis, waveguides of a width between 300 and 1000nm in steps of 25nm were considered. The experiments presented are based on waveguides as described in Section 3.2.

#### 2.1.1. Surface Topology

For the experimental part of this work, atomic layer deposition (ALD) layers were chosen, as they are known to form homogeneous and conformal layers in a reproducible way [15,16].

To reduce the need for conformal growth and make the sensor usable for a wider range of layers, different waveguide geometries were examined theoretically. The difficulties during layer growth can arise from the difference between horizontal and vertical surfaces of the waveguide as well as effects that occur at the edges between those surfaces. These effects could be included in the simulations. This requires very precise knowledge about the layer formation process.

In addition to the geometries according to Figure 2a,b, we also theoretically investigated alternative waveguide geometries, as depicted in Figure 2c–f. The proposed geometry (c) takes care of the difficulties at the lower, concave edge between the side wall of the waveguide and its surroundings, leading to a slight increase of sensor sensitivity beyond the values obtained for conventional nanowires.

The proposed waveguide structures (d)–(f) solve the problem between horizontal and vertical surfaces at the cost of a reduced sensor sensitivity. Here the covered nanowire and the covered nanorib only consider the field at the top surface of the waveguide for sensing (Figure 2e,f). This surface can be produced at very high quality using chemical-mechanical polishing (CMP). The sandwiched nanowire (Figure 2d) uses the field at the waveguide sidewalls and might suffer from the side wall roughness caused by lithography and etch steps during the sensor production.

#### 2.1.2. Higher Order Modes

Some of the waveguides considered in the theoretical part support multiple quasi-TE modes. For the purposes of this paper, only their fundamental modes were considered. Coupling between different modes can lead to an ambiguity in the results of the measurements.

For racetrack ring resonators as depicted in Figure 1 coupling between the waveguide modes can occur at the transitions between the bended and the straight parts of the ring resonator. This can be avoided by either using circular ring resonators or by a distortion of the bends to compensate for the mode mismatch and prevent coupling as described in [17]. Additionally, the access waveguide can be tapered down before and after the coupling with the ring resonator to a width where it supports only a single quasi-TE mode. These filter regions ensure that only the contributions of the fundamental mode of the access waveguide are measured.

### 2.2. Sensor Chips

The sensor chips used nanowire and nanorib waveguide structures etched into the 220nm top silicon layer of a SOI wafer according to Figure 2a,b. Sets of 10 nominally identical ring resonators were coupled in a serial manner to a common access waveguide. For optical coupling to and from the chip, grating couplers were used. They were etched in the same process as the nanorib waveguides.

The ring resonators were used to translate the change in effective index caused by the presence of the sample layer into a change in resonance frequency that then could be measured in the transmission spectrum of the ring resonator.

**Figure 3:**
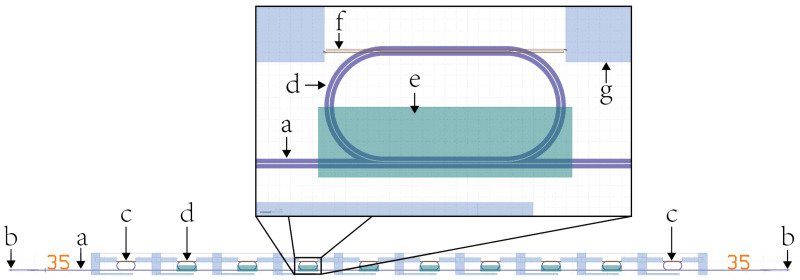
Chip design of the waveguides used in the experimental part of this work. The optical waveguides (trenches (a) shown in purple) start and end in grating couplers (b). Of the 10 racetrack ring resonators ((c) and (d), also in purple) coupled to the waveguide, eight have opened areas ((e), green) to allow for the sample interaction. The first and last ring resonators (c) are used for reference measurements. Electrical contacts ((g), blue) connect to heating wires ((f), brown) used for modulating individual ring resonators.

A passivation including a metallization layer embedded in ${\mathrm{SiO}}_{2}$ contained heating wires positioned above part of each ring resonator. This allowed for the thermo-optical modulation of each ring resonator (marked *f* in Figure 3). The passivation was opened such that half of each ring resonator was accessible for use as a sensing area. Two of the otherwise identical 10 ring resonators per access waveguide were completely covered by ${\mathrm{SiO}}_{2}$ passivation to act as a reference. See Figure 3 for the layout of such a waveguide.

### 2.3. Measurement System

To determine the resonance wavelength of the ring resonators, transmission spectra were obtained by sweeping a tunable laser source (TLS) (relative wavelength accuracy ±7pm) through the desired wavelength range and recording the transmission for each wavelength using a power meter (PM).

Due to the design of the sensor chip, up to ten ring resonators were coupled to a single access waveguide. For measurements, it is necessary to extract the information of a single ring resonator [18]. For this purpose, the ring resonator under testing was thermally modulated at a frequency of 1kHz and a lock-in amplifier was used to extract the signals corresponding to this ring. This was done for each wavelength [19].

**Figure 4:**
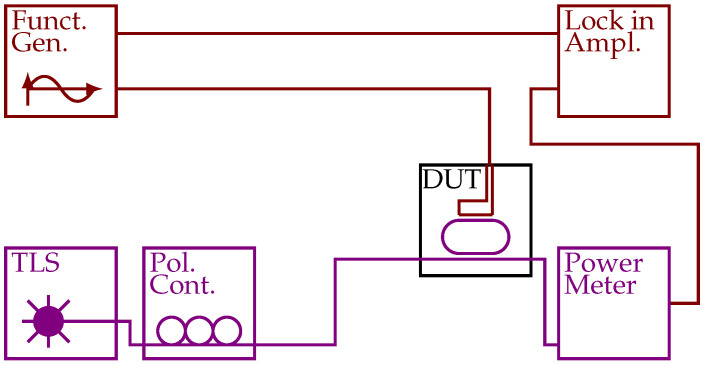
Experimental setup used for the measurement of the resonance wavelengths of a ring resonator. Using single mode fibers and grating couplers, light from a TLS was coupled into an access waveguide and out to a power meter. A function generator supplying a sinusoidal signal and a lock-in amplifier locked to the double frequency of the sinusoidal were used to extract the signal of a single ring resonator. The SOI chip with the ring resonator is labeled device under test (DUT); optical connections are indicated in purple and electrical connections in red.

Figure 4 shows a sketch of the experimental setup used for obtaining the resonance wavelength of the ring resonators. A TLS and a power meter form a standard setup for measuring transmission spectra. As multiple ring resonators were coupled to a single access waveguide (see Figure 3), a method for obtaining information about a single ring was required. For that, the ring resonator of interest was thermooptically modulated using a function generator and a lock-in amplifier was locked to the double frequency of the modulation frequency. This allows for the extraction of a signal proportional to the derivative of the transmission spectrum of the modulated ring resonator but not the other ring resonators coupled to the same access waveguide. For more information about this setup, see [18,19].

### 2.4. Simulations

For both the theoretical results and for the analysis of the experimental data, the effective refractive index of waveguide structures needed to be determined. This was done via eigenmode analysis using the tool JCM-wave [20,21].

## 3. Results and Discussion

### 3.1. Theoretical Results

For the theoretical part of this work, the influence of thin layers of a material deposited onto the sensor surface on the effective index of the fundamental mode of waveguides with various geometries was determined. The influences of the layer thickness, the refractive index and the measurement wavelength were determined. From those, the sensitivity of a ring resonator at a given waveguide geometry to changes in the layer refractive index and layer height was calculated using:

(1) $$\begin{array}{c} S_{i,n}=\frac{d\lambda }{dn_{l}}=\frac{\lambda }{N_{eff,i}\left(\lambda ,n_{l},h_{l}\right)}\frac{dn_{eff,i}\left(\lambda ,n_{l},h_{l}\right)}{dn_{l}} \end{array}$$

(2) $$\begin{array}{c} S_{i,h}=\frac{d\lambda }{dh_{l}}=\frac{\lambda }{N_{eff,i}\left(\lambda ,n_{l},h_{l}\right)}\frac{dn_{eff,i}\left(\lambda ,n_{l},h_{l}\right)}{dh_{l}} \end{array}$$

(3) $$\begin{array}{c} N_{eff,i}\left(\lambda ,n_{l},h_{l}\right)=n_{eff,i}\left(\lambda ,n_{l},h_{l}\right)-\lambda \frac{dn_{eff,i}\left(\lambda ,n_{l},h_{l}\right)}{d\lambda } \end{array}$$

with the effective index $n_{eff,i}\left(\lambda ,n_{l},h_{l}\right)$, the group effective index $N_{eff,i}\left(\lambda ,n_{l},h_{l}\right)$, the layer refractive index $n_{l}$, height $h_{l}$, the wavelength λ, layer refractive index sensitivity $S_{i,n}$ and layer height sensitivity $S_{i,h}$. i=1…j is the index for the ring resonators that compose the sensor; *j* is the number of ring resonators with different waveguide widths used in the sensor. For the simulations, j=2…29 different waveguide widths were used. In the experiment there were j=3 rings with different waveguide geometries.

Linearizing the response of each sensor around a set of layer properties, in this case $n_{l,0}=2.433$ and $h_{l,0}=22\;nm$ with the corresponding resonance wavelengths $\lambda_{i,0}$, gives a set of linear equations. This set has as many (*j*) equations as sensors with different waveguide geometries or widths are used and two unknowns, the layer refractive index $n_{l}$ and height $h_{l}$.

(4) $$\begin{array}{ccc} \left[\begin{array}{c} \lambda_{1}\\ \lambda_{2}\\ \cdots \end{array}\right]-\left[\begin{array}{c} \lambda_{1,0}\\ \lambda_{2,0}\\ \cdots \end{array}\right]=\left[\begin{array}{c} \delta \lambda_{1}\\ \delta \lambda_{2}\\ \cdots \end{array}\right] & = & \left[\begin{array}{cccc} & S_{1,n} & S_{1,h} & \\ & S_{2,n} & S_{2,h} & \\ & \cdots & \cdots \end{array}\right]\;\cdot \;\left(\left[\begin{array}{c} n_{l}\\ h_{l} \end{array}\right]-\left[\begin{array}{c} n_{l,0}\\ h_{l,0} \end{array}\right]\right) \end{array}$$

(5) $$\begin{array}{ccc} \;\;\;\;\;\;\;\;\;\;\;\;\left[\begin{array}{c} \delta \lambda_{1}\\ \delta \lambda_{2}\\ \cdots \end{array}\right] & = & \left[\begin{array}{cccc} & S_{1,n} & S_{1,h} & \\ & S_{2,n} & S_{2,h} & \\ & \cdots & \cdots \end{array}\right]\;\cdot \;\left[\begin{array}{c} \delta n_{l}\\ \delta h_{l} \end{array}\right] \end{array}$$

with the resonance wavelength $\lambda_{i}$ and the wavelength error $\delta \lambda_{i}$ for waveguide *i*; and $\delta n_{l}$ and $\delta h_{l}$ for the layer refractive index error and height error, respectively.

If more than two different waveguide geometries are used, the system of equations is overdetermined and does not have an exact solution. It can still be solved numerically such that the mean square of the errors in $\lambda_{i}$ is minimized.

#### 3.1.1. Measurement Uncertainty

One cause of measurement uncertainty that is inherent to the use of ring resonator sensors comes from the accuracy of the wavelength measurements. A Monte Carlo method was applied to determine the influence of independent random errors occurring for the measurement of each wavelength shift. By numerically inverting the set of linear equations given in Equation (4) for different sets of measurement deviations $\delta \lambda_{i}$ drawn from a standard deviation with a mean of 0pm and a standard deviation of 10pm, the influence of those deviations on the measured system variables ($\delta n_{l}$ and $\delta h_{l}$) defined as the mean absolute of the deviations was determined.

For each of the waveguide geometries, this was done for sets of two to 29 different waveguide widths chosen from the range of simulated waveguide widths of 300,325,…1000nm. Uncorrelated measurement errors for the wavelength measurements $\delta \lambda_{1}\ldots \delta \lambda_{j}$, each as an independant random variable drawn from the same distribution, were assumed. In case of less than 10,000 possible selections of waveguide widths for a given number *j* of waveguides to be selected, all possible selections were examined. In cases with more possible combinations, 10,000 random ones were sampled. For each number of different waveguide widths *j*, the combination of waveguides that lead to the smallest measurement uncertainties was considered.

**Figure 5:**
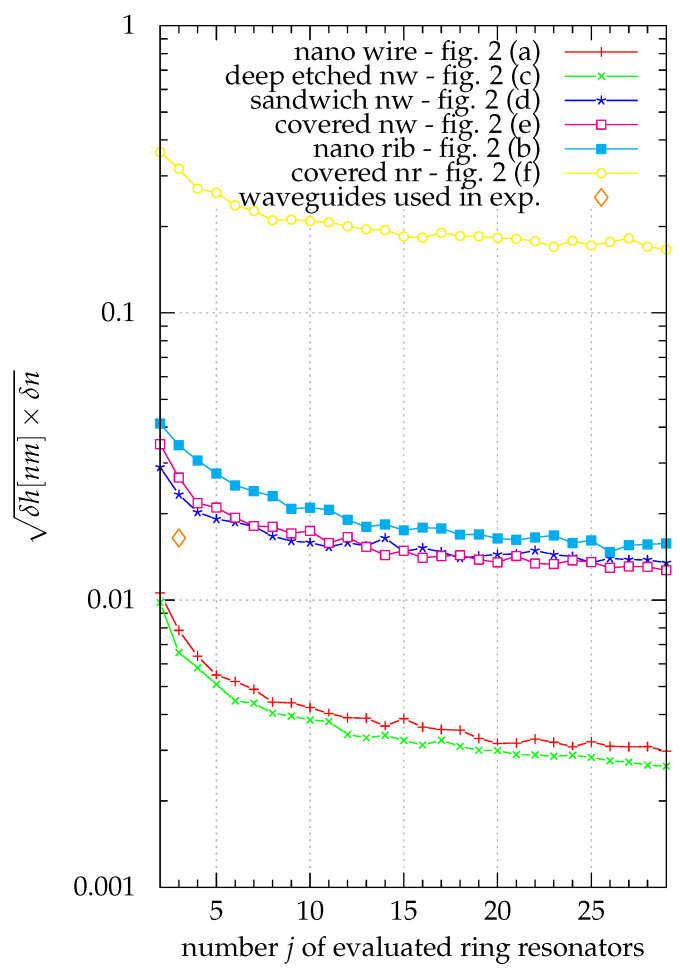
The geometrical mean of the precision in refractive index and in layer thickness (in nano meters) as a figure of merit for the sensor system is plotted over the number of different waveguides used for different waveguide geometries. The only uncertainty considered is the wavelength accuracy of the resonance wavelengths. Production errors for the geometrical waveguide properties are neglected to show the theoretical limitations of the method. When not all waveguide widths were used, the combination with the lowest figure of merit was used.

The geometric mean of $\delta n_{l}$ and $\delta h_{l}$ in nm is introduced as a figure of merit (FOM) to combine the uncertainties for both the layer height and refractive index. This allows for the simultaneous optimization for both factors and an easy comparison of the different sensors. This FOM is calculated as $FOM=\sqrt{\delta h[nm]\;\cdot \;\delta n}$ using $\delta n_{l}$ and $\delta h_{l}$ obtained from the montecarlo simulation using the numerically inverted set of linear equations. The simulation results are given in Figure 5 using this figure of merit.

As a result, adding additional complexity by including more ring resonators using waveguides of different width increased the sensitivity. The best result was obtained using all 29 over-etched nanowire waveguides. Here the theoretical limit for the accuracy of the refractive index measurement and the height measurement are 0.0006 and 0.01nm, respectively. A more realistic case where three nanowire waveguides (width 300nm, 625nm and 925nm) are used gave 0.002 and 0.04nm, respectively. A good example for a sensor consisting of three covered nanowires (width 300, 775 and 825nm) leads to 0.004 and 0.1nm, respectively.

The predictions for the combination of the three types of waveguide available for the experimental part (see Table 1, target values) are accuracies of the refractive index measurement and the height measurement of 0.003 and 0.08nm, respectively.

#### 3.1.2. Production Tolerances

To reach the optimum performance only limited by the precision of the wavelength measurement and the homogeneity of the examined layer the waveguide cross section of the waveguide geometries used needs to be known with a high precision. In the case of a nanowire waveguide this includes knowing the waveguide width and height, the refractive index of all materials used and the sidewall angle to a precision not necessary for other applications. For a nanorib waveguide this would additionally include the etch depth or slab height.

For most of the waveguide properties there are methods to precisely control or measure them. The top Silicon layer height of a wafer that defines the waveguide height for example can be measured using ellipsometry. Here the waveguide width was examined as a property that is much harder to precisely control or measure. Systematic errors can be caused during the photolithographic or the etching process and can depend on the position on the wafer.

**Figure 6:**
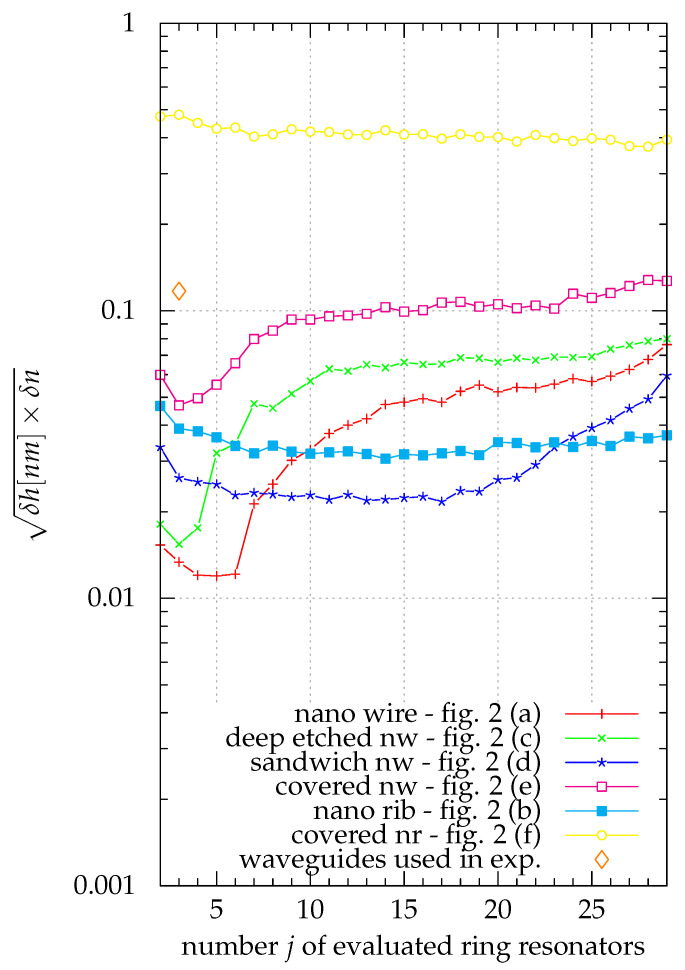
The geometrical mean of the precision in refractive index and in layer thickness (in nano meters) as a figure of merit for the sensor system is plotted over the number of different waveguides used for different waveguide geometries. The values here consider both the uncertainties caused by an offset of all waveguide widths by ±1nm as well as the wavelength accuracy of the resonance wavelength measurements. When not all waveguide widths were used, the combination with the lowest figure of merit was used. Comparing the results to Figure 5, it can be seen that geometrical variations of the waveguides can easily dominate the measurement uncertainty.

The influence of the width of all waveguides being off by ±1nm was evaluated by simulating the sensor system with an offset waveguide width and analyzing the results using Equation (4) for the system as designed. This was repeated for different random offsets drawn from a normal distribution with a standard deviation of 1nm whilst also applying the same random deviations as above to the wavelength measurements. The results are shown in Figure 6.

According to Figure 6, the error in waveguide width becomes the limiting factor for all waveguide geometries examined. The nanowire waveguides still show the best performance. Unlike the results where only statistical errors of the measured wavelength were considered, adding data from more of the different simulated waveguides did not necessarily improve the results anymore.

Considering the systematic waveguide width error the best result, 0.002 and 0.06nm for the accuracy of the refractive index measurement and the height measurement respectively, was obtained with five nanowire waveguides of the width 300nm, 400nm, 425nm, 475nm and 500nm. For covered nanowire sensors 0.009 and 0.2nm was achieved with waveguide widths of 300nm, 375nm and 400nm. For the combination of the three types of waveguide used in the experiments the accuracy became 0.02 and 0.6nm assuming the same systematic width error for all three waveguide types disregarding the fact that nanowire waveguides and nanorib waveguides were produced in different processes.

#### 3.1.3. Selection of Waveguides

From Figure 5 and Figure 6 it can be seen that in general waveguide types that have a stronger field overlap with the area deposited layer lead to more sensitive sensor systems. For the classical waveguide types nanowire and nanorib this means that the nanowire is more sensitive.

If the systematic waveguide width error is considered (see Figure 6) sensors built from nanowire sensors are also the most sensitive sensors. The only reason to choose any of the modified geometries over nanowire sensors is the deposition of the layer to be examined. If it is not feasible to deposit a sample layer homogeneously on the structured surface of a sensor chip using nanowire waveguides the proposed new waveguide geometries can be beneficial. Here the sandwich nanowire and the covered nanowire can be used to provide flat vertical and horizontal surfaces in the interaction area, respectively. This comes at a cost in sensitivity of 3dB and 6dB, respectively.

The covered nanorib can be disregarded. It has the lowest field overlap with the deposited layer and is outperformed by a solid margin by all other proposed waveguide geometries.

### 3.2. Experimental Results

**Figure 7:**
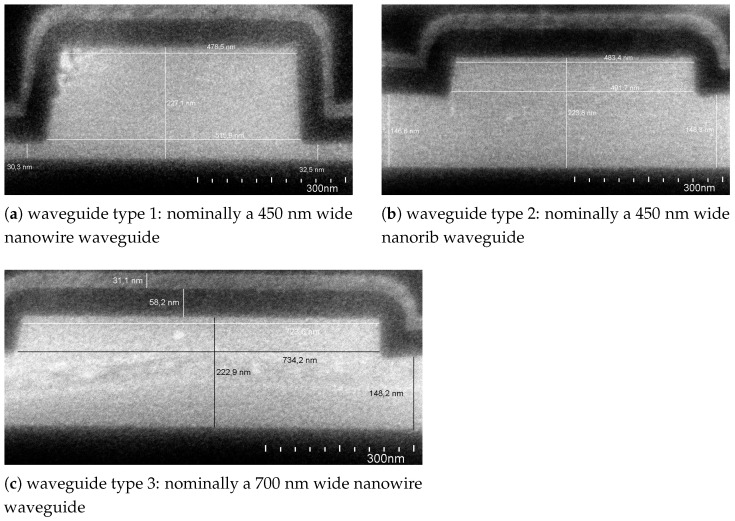
SEM images of the waveguide types used with the measured dimensions. Waveguides of type 1, designed as nanowire waveguides, were not etched down to the silica as planned, but still had a slab height of ≈31.4nm. The waveguides showed slightly wider width compared to the design values.

As a first test of the theoretical results a sensor chip containing three types of waveguides of different dimensions (see Figure 7) was coated with an ALD layer of ${\mathrm{TiO}}_{2}$. For each of the waveguide geometries the resonance wavelengths of one ring resonator were measured before and after the deposition of the ALD layer. The measurement results were analyzed in comparison with simulations considering the real waveguide geometry as measured via scanning electron-beam microscopy (SEM).

#### 3.2.1. ALD Layer Deposition

Plasma enhanced atomic layer deposition (PE-ALD) was applied to deposit ${\mathrm{TiO}}_{2}$ layers on the structured surfaces of the sensors using a PE-ALD system by SENTECH Instruments equipped with a remote conductively coupled plasma source. PE-ALD is an advanced method of extending the capabilities of ALD by applying radical gas species as precursor co-reactant during the deposition process [22]. A layer thickness of around 23nm was produced at a substrate temperature of $270{\;}^{\circ }C$. $N_{2}$ (40sccm) was used as carrier gas for the $70{\;}^{\circ }C$ heated titanium precursor Titanium isopropoxide (TTIP), while atomic oxygen was generated by the plasma source with a constant oxygen flow rate of 200sccm controlled by a mass flow controller. The plasma source was operated in a pulsed mode with a power of 200W. The purging periods in between the PE-ALD steps were typically 5 and 2s after the TTIP and plasma pulse, respectively. The process pressure was 20Pa.

#### 3.2.2. Data Evaluation

To analyze the measured wavelength shifts simulations were done for both the state of the sensor before as well as after the deposition of the layer. For these simulations the expected layer thickness and refractive index were used as starting values. This resulted in expected wavelength shifts $\Delta \lambda_{i}^{s}=\lambda_{i}^{s}-\lambda_{i}^{s,nl}$ that were compared to the measured wavelength shifts $\Delta \lambda_{i}^{m}=\lambda_{i}^{m}-\lambda_{i}^{m,nl}$ with $\lambda_{i}^{s}$ and $\lambda_{i}^{s,nl}$ for the measured resonance wavelength of sensor *i* before and after the deposition of the layer and $\lambda_{i}^{m}$ and $\lambda_{i}^{m,nl}$ for the simulated resonance wavelength of sensor *i* with and without the layer. The differences were used to calculate correction factors for layer thickness and refractive index by linearizing $\lambda_{i}^{s}$ around the expected values for $n_{l}$ and $h_{l}$ using Equation (5):

(6) $$\begin{array}{ccc} \left[\begin{array}{c} \Delta \lambda_{1}^{s}\\ \Delta \lambda_{2}^{s}\\ \Delta \lambda_{3}^{s} \end{array}\right] & = & \left(\left[\begin{array}{c} \lambda_{1}^{s}\\ \lambda_{2}^{s}\\ \lambda_{3}^{s} \end{array}\right]-\left[\begin{array}{c} \lambda_{1}^{s,nl}\\ \lambda_{2}^{s,nl}\\ \lambda_{3}^{s,nl} \end{array}\right]\right) \end{array}$$

(7) $$\begin{array}{ccc} & \;\;\;\;\;\;\;\;\;\;\;\;\;\;\;\;\;\;\;\;\;\;\;\;\;\;\;\;\;\;\;\;\;\;\;\;\;\;\;\;\;\;\;\;\;\;\;\;\;\;\;\;\;\;\;\;\;\;\;= & \left(\left[\begin{array}{c} \lambda_{1,0}^{s}\\ \lambda_{2,0}^{s}\\ \lambda_{3,0}^{s} \end{array}\right]+\left[\begin{array}{cccc} & S_{1,n} & S_{1,h} & \\ & S_{2,n} & S_{2,h} & \\ & S_{3,n} & S_{3,h} \end{array}\right]\;\cdot \;\left[\begin{array}{c} \delta n_{l}\\ \delta h_{l} \end{array}\right]-\left[\begin{array}{c} \lambda_{1}^{s,nl}\\ \lambda_{2}^{s,nl}\\ \lambda_{3}^{s,nl} \end{array}\right]\right) \end{array}$$

The calculations were done in analogy with the theoretical part by numerically inverting the overdetermined system of linear equations and calculating the corrections $\delta n_{l}$ and $\delta n_{h}$ to the expected layer thickness and refractive index respectively.

The same set of simulations was applied for the new, corrected layer properties determining new values for $\lambda_{i}^{s}$, $S_{i,n}$, $S_{i,n}$ and ultimately for $\delta n_{l}$ and $\delta n_{h}$. This was repeated until the changes in the layer properties between two simulation steps become less then 0.01 for the layer refractive index and less than 0.01nm for the layer height.

#### 3.2.3. Production Tolerances

In the theoretical part of this work it has already been shown that small deviations in the waveguide width can have a huge impact on the accuracy of the measurement. As the geometrical dimensions of the produced waveguides can vary, even within a sample of sensor chips produced together on the same wafer it is not possible to just use the design geometry in the simulations required for data evaluation. Instead the waveguide profiles of the sensor chip were examined using SEM and the dimensions extracted from the SEM images were used for the simulations.

A comparison of the waveguide dimensions as measured via SEM and the corresponding design values is given in Table 1 and the SEM images are given in Figure 7. For the parts of the ring resonator covered by ${\mathrm{SiO}}_{2}$, the thin silicon nitride layer that serves as an etch stop in the production process was also considered.

It is possible to replace the need for an SEM measurement with a calibration step using layers with known thickness and refractive index.

#### 3.2.4. Ambiguity of Spectral Data

Due to the small free spectral range (FSR) of the ring resonators used (≈2.1nm for waveguide type 1 and ≈1.2nm for waveguide type 2 and 3) and the large expected spectral shifts of up to 18nm, the measurement results for the resonance wavelength shift of a single ring resonator can be ambiguous by multiples of the FSR of that ring resonator.

To narrow down the number of possible solutions for the resonance shift of each ring resonator, knowledge about the deposited layer was used. From experience with the ALD process and ellipsometric control measurements, it was known that the layer height has to be in the range of 20…26nm. This left two potential values for the wavelength shift for each of the three ring resonators evaluated for the experiment. Each of the eight resulting combinations of measurement results was evaluated according to the procedure given above. The evaluation procedure minimizes the root mean squared (RMS) of the wavelength errors in the overdetermined system of equations:

(8) $$RMS=\sqrt{\sum_{i=1}^{3}\frac{{\left(\Delta \lambda_{i}^{m}-\Delta \lambda_{i}^{s}\left(h_{l},n_{l}\right)\right)}^{2}}{3}}$$

This RMS was used as a measure for the quality of the solution. As the system of linear equations is overdetermined and there are measurement uncertainties, the RMS that is minimized by the optimization process will generally not reach zero. It only gets small for combinations of measured wavelength shifts that can be described by the same combination of $n_{l}$ and $h_{l}$. This is used to select between the remaining eight combinations. The lowest value of RMS=33pm was achieved for the combination of end wavelengths given in Table 2.

The second to lowest value for the RMS that was found is 121pm and would have led to a result of 25.7nm and 2.40 for the layer thickness and refractive index respectively.

#### 3.2.5. Final Result

Taking into account all of the effects mentioned in the previous sections, the data from the experiment are evaluated as described above. The final result gives $n_{layer}=2.39\pm 0.02$ and $h_{layer}=22.3\pm 0.6\;nm$ for the refractive index and the thickness of the deposited ALD layer respectively. Table 3 compares these results with the target values of the ALD layer.

For the uncertainties the values from the theoretical part are used as an upper limit. Due to the use of SEM metrology combined with a calibration sample systematic errors in the dimensions of the waveguides are assumed to be smaller than 1nm.

## 4. Conclusions

A novel, evanescent field-based sensor for the simultaneous measurement of the thickness and the refractive index of a layer was demonstrated. The high sensitivity with theoretical limits of 0.0006 and 0.01nm for the accuracy of the refractive index measurement and the height measurement, respectively, was determined, and the initial experiments were successfully performed to verify the theoretical results.

A limitation of the experiments presented in this work is the use of readily available waveguide geometries not optimized for this task. Further experiments using waveguide geometries selected according to the theoretical results in this work are necessary to show the full potential of the sensor. The sensitivity can be increased by selecting more suitable sets of waveguide widths which does not require any change to the processes used. The applicability to a wider variety of sample layers can be increased by developing processes to reliably produce some of the newly proposed geometries that provide a flatter surface in the sensing region. An optimized sensor system will use ring resonators of a higher FSR to remove or at least reduce the ambiguity of the wavelength measurements. Additionally, the whole surface of the ring resonator can be made accessible. This is expected to increase the sensitivity of the ring resonators and reduce the complexity of the data analysis.

The experimental procedure described requires SEM measurements to gain a precise knowledge of the waveguide geometries. Such measurements are destructive, and their precision is the limiting factor of the method. It is possible to overcome this need by using well known calibration layers. For example, a thin (compared to the penetration depth of the optical field) ALD layer can be deposited on the chip before using it for the actual measurement. By measuring the wavelength shifts caused by the deposition of this calibration layer, information about production tolerances can be gained and used to compensate for them [23].

While the theoretical results rival those achievable by ellipsometry, further work is required to ensure a precise knowledge of the waveguide dimensions to reach this performance. The advantages of this sensor are a high integration and the ability for the simultaneous measurement of multiple parameters. This makes it a good candidate for in situ monitoring of the growth of thin film layers.

## Figures and Tables

**Table 1:** Comparison of design values and values measured with scanning electron-beam microscopy (SEM) for the geometrical waveguide properties.

Waveguide Type	Waveguide Width		Etch Depth	
	Design	SEM	Design	SEM
waveguide type 1	450.0nm	478.5nm	220.0nm	193.2nm
waveguide type 2	450.0nm	483.4nm	70.0nm	76.9nm
waveguide type 3	700.0nm	723.6nm		
Silicon Layer Height	220.0nm	224.6nm		

**Table 2:** Resonance wavelength before and after the deposition of the atomic layer deposition (ALD) layer.

Waveguide Type	Start Wavel.	End Wavel.	Wavel. Shift
waveguide type 1	1549.22nm	1562.87nm	13.65nm
waveguide type 2	1548.68nm	1558.00nm	9.32nm
waveguide type 3	1548.70nm	1556.00nm	7.30nm

**Table 3:** Measured layer properties compared to the target values of the ALD process. The uncertainties of the target values are unknown.

	Measured	Design Value (from ALD Process)
layer refractive index $n_{layer}$	2.39±0.02	2.43
layer thickness $h_{layer}$	22.3±0.6nm	23nm

## Abbreviations

The following abbreviations are used in this manuscript:

ALD	atomic layer deposition
CMP	chemical-mechanical polishing
DUT	device under test
FOM	figure of merit
FSR	free spectral range
PE-ALD	plasma enhanced atomic layer deposition
PM	power meter
RMS	root mean squared
SEM	scanning electron-beam microscopy
SOI	silicon on insulator
TE	transverse electric
TLS	tunable laser source
TM	transverse magnetic
